# Intramucosal adenocarcinoma of the ileum originated 40 years after ileosigmoidostomy

**DOI:** 10.1186/1477-7819-7-41

**Published:** 2009-04-21

**Authors:** Shinichi Sameshima, Shigeru Tomozawa, Shinichiro Koketsu, Toshiyuki Okada, Hideyo Miyato, Misa Iijima, Masaru Kojima, Toshio Kaji

**Affiliations:** 1 Department of Surgery, Hitachi Yokohama Hospital, 550 Totsuka-cho, Totsuka-ku, Yokohama, Kanagawa 244-0003, Japan; 2 Department of Surgery, Gunma Cancer Center, 617-1 Takabayashi-nishi, Ota, Gunma 373-8550, Japan; 3 Department of Pathology, Gunma Cancer Center, 617-1 Takabayashi-nishi, Ota, Gunma 373-8550, Japan

## Abstract

**Background:**

Small bowel adenocarcinomas (SBAs) are rare carcinomas. They are asymptomatic and usually neither endoscopy nor contrast studies are performed for screening

**Case presentation:**

A 72-year-old Japanese male had a positive fecal occult blood test at a regular check-up in 2006. He suffered appendicitis and received an ileosigmoidostomy in 1966. A colonoscopy revealed an irregular mucosal lesion with an unclear margin at the ileum side of the anastomosis. A mucosal biopsy specimen showed adenocarcinoma histopathologically. Excision of the anastomosis was performed for this patient. The resected specimen showed a flat mucosal lesion with a slight depression at the ileum adjacent to the anastomosis. Histological examination revealed a well differentiated intramucosal adenocarcinoma (adenocarcinoma in situ). Immunohistological staining demonstrated the overexpression of p53 protein in the adenocarcinoma.

**Conclusion:**

Adenocarcinoma of the ileum at such an early stage is a very rare event. In this case, there is a possibility that the ileosigmoidostomy resulted in a back flow of colonic stool to the ileum that caused the carcinogenesis of the small intestine.

## Background

Small bowel adenocarcinomas (SBAs) are rare carcinomas. They are asymptomatic and usually neither endoscopy nor contrast studies are performed for screening. Most of SBAs are detected at the advanced stage. Early stage SBAs are extremely rare cases.

We report a case of an intramucosal adenocarcinoma (adenocarcinoma in situ) of the ileum mucosa an ileosigmoidostomy. A few cases with adenocarcinoma in situ of small bowel have been reported [1]. There is no report of an adenocarcinoma of the ileum following the ileocolonostomy in the literature.

## Case presentation

A Japanese male suffered severe appendicitis and received an ileosigmoidostomy without appendectomy in 1966. A prostatectomy was performed for benign prostate hypertrophy at the age of 67. He also received medical treatment for hypertension. A regular check-up in August 2006, when the patient was 72 years of age, revealed a positive fecal occult blood test. A colonoscopy was conducted by his family practitioner and an irregular mucosal lesion with an unclear margin was detected at the ileum mucosa adjacent to the anastomosis. Histological examination of a mucosal biopsy revealed a well differentiated adenocarcinoma. He was then referred to our hospital in October 2006.

**Figure 1:**
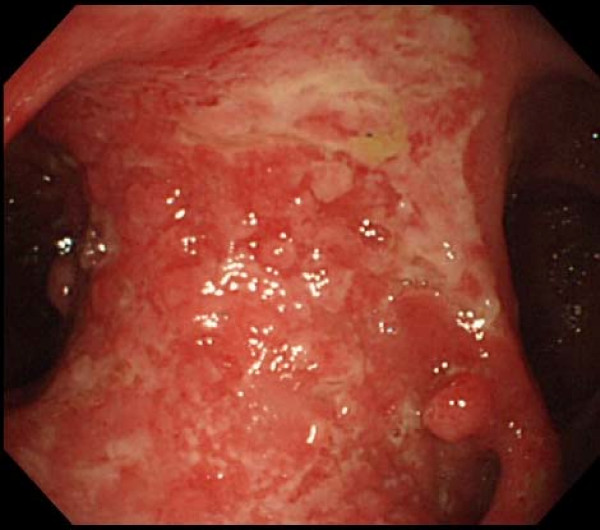
**Colonoscopic findings from the colon side, showing a wide irregular mucosal lesion with white mucus at the ileum**.

He showed no abdominal complaints upon admission. Physical examination showed no abnormal findings other than the operation scar from the bypass operation. Carcinoembryonic antigen (CEA) and carbohydrate antigen 19-9 (CA 19-9) were within the normal range. We conducted another colonoscopy and identified an ileo-colonic anastomosis 28 cm from the anal verge. It showed an irregular mucosal surface with a diameter of 4 cm at the ileum (Fig. 1). Histological analysis of the mucosal biopsy showed a well differentiated adenocarcinoma of the ileum. A small bowel series and a large bowel series revealed an ileosigmoidostomy in the right lower abdomen. Abdominal computed tomography showed an area with mild thickening in the intestine below the right lower abdominal wall. There was no finding of lymph node swelling or liver metastases.

Surgical exploration was undertaken with the tentative diagnosis of carcinoma of the ileum. The ileosigmoidostomy was identified at the oral side of the ileum, 100 cm from the ileocecal valve. No definite tumor was detected at that anastomotic site. The anastomosis with 7 cm of ileum and 20 cm of sigmoid colon were resected collectively. The ileum was reconstructed by functional-end-to-end anastomosis and the sigmoid colon was reconstructed by the double stapling technique.

**Figure 2:**
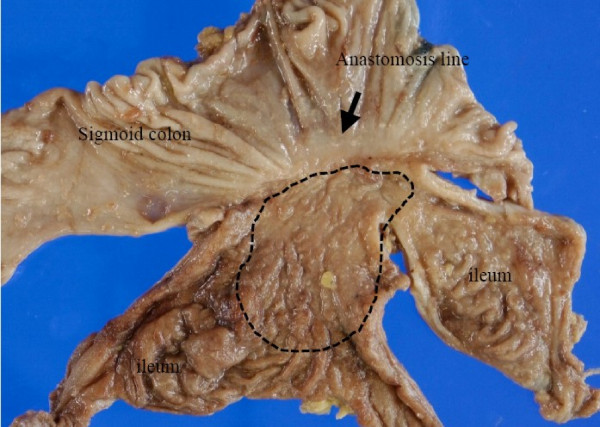
**The resected specimen showing the small bowel and sigmoid colon, including the anastomosis (black arrow)**. The flat lesion was widely spread around the ileum side of the anastomosis, but not infiltrating into the sigmoid colon. Adenocarcinoma was observed in the area surrounded by dots.

**Figure 3:**
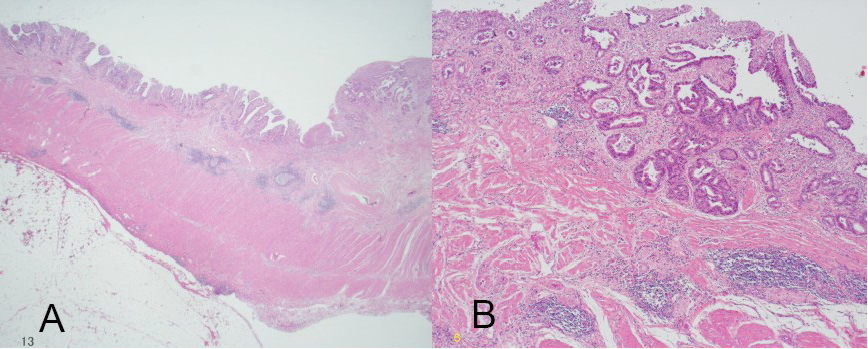
**Well differentiated adenocarcinoma in the mucosal layer (A: hematoxylin and eosin, ×10, B: hematoxylin and eosin, ×100)**.

**Figure 4:**
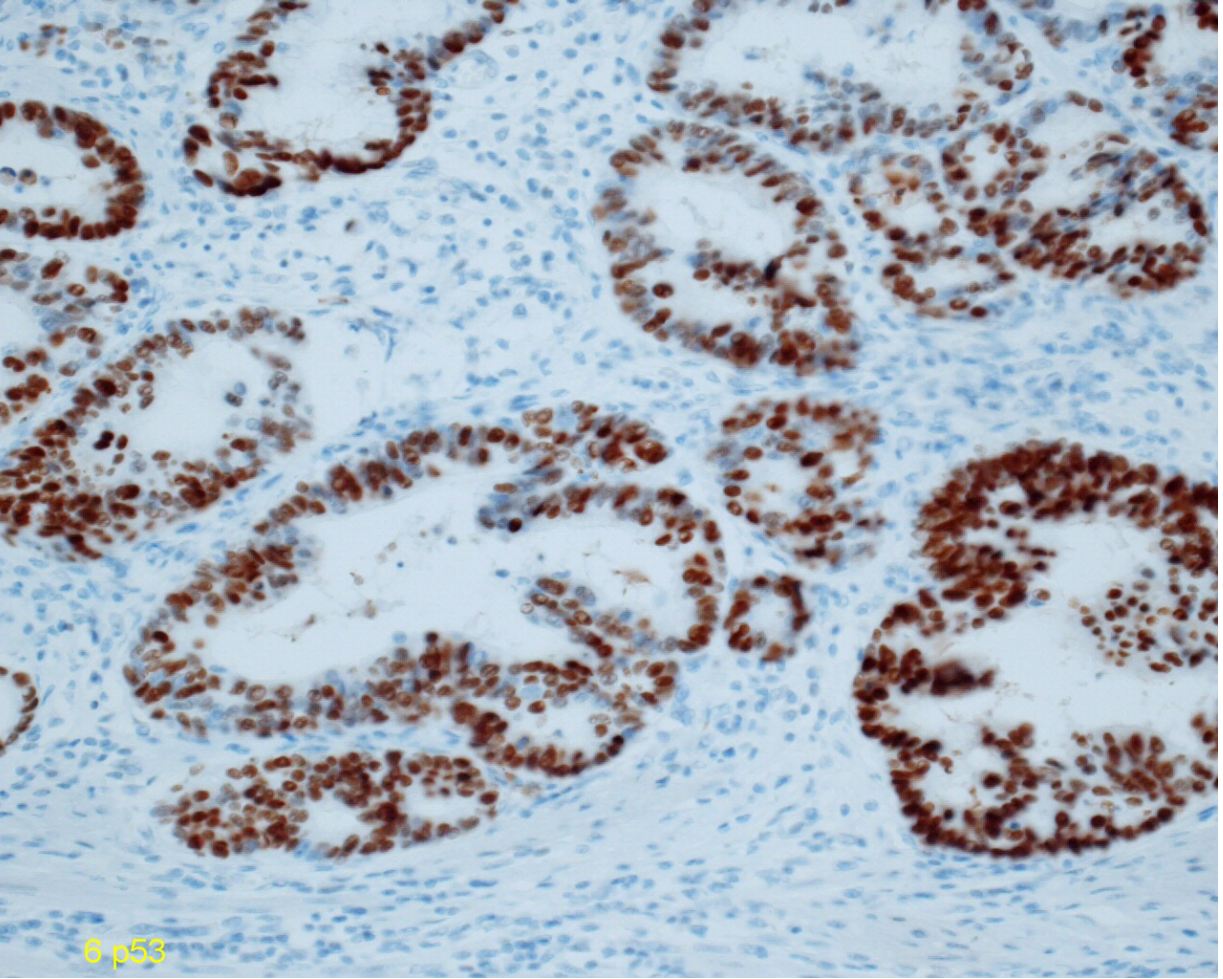
**Over-expression of p53 protein was observed in the adenocarcinoma immunohistochemically**.

The resected specimen showed a flat mucosal lesion with a slight depression at the ileum adjacent to the anastomosis (Fig. 2). Histological examination of the specimen revealed intramucosal adenocarcinoma (Tis). It was detected in the ileum mucosa and not at the sigmoid colon side (Fig. 3A, B). Immunohistological staining of p53 protein was performed for the resected specimen with carcinoma using D0-7 (Dako Cytomation, Inc. Carpinteria, CA, USA) as the first antibody and iVIEW DAB Detection kit (Ventana Medical Systems, Inc. Tucson, AZ, USA). Over-expression of p53 protein was observed at the dysplastic gland of the ileum (Fig. 4).

We got an informed consent from the patient to use the patient's data for a case report.

## Discussion

SBAs accounted for only 2.1% of new cases of all gastrointestinal malignancies in 2005 in the United States [1-3]. Further, the majority of SBAs occur in the duodenum. In fact, SBAs elsewhere than the duodenum are rare tumors, despite this area comprising more than 90% of the surface area of the gastrointestinal tract [4]. A number of explanations for this have been proposed, including low bacterial content, neutral or alkaline environment, presence of copious lymphoid tissue with high levels of IgA and enzymes to inhibit carcinogens, and a fast transit time which reduces the exposure to carcinogens [5].

SBAs are diagnosed at a more advanced stage. Early stage adenocarcinomas in the small intestine are extremely rare entities. After surgical resection, only 0–10% of SBAs are found in stage T1 and 0–3% in stage Tis [6]. Clinically, it is extremely difficult to detect SBAs in the early stage. They tends to be asymptomatic and usually neither small bowel endoscopy nor contrast studies are performed for screening, except for patients with familial adenomatous polyposis or Crohn's disease[7]. Indeed, most SBAs are diagnosed at advanced stages and adenocarcinomas at the early stage are rarely-detected entities.

Inflammation of the intestine is thought to cause a precancerous lesion in intestinal organs. Ulcerative colitis patients with long-term inflammation often show colorectal dysplasia which leads to carcinoma [8,9]. Crohn's disease patients with a long history of inflammation are also reported to develop carcinomas in the small intestine and colorectum [10,11]. It was reported that p53 protein overexpression was detected at the dysplasia and the adenocarcinoma associated the ulcerative colitis and Crohn's disease [12-14]. This case showed the flat type of adenocarcinoma with p53 positive cells. Some reports have demonstrated the development of malignancy at the ileal pouch after the total proctocolectomy for the ulcerative colitis patients [15,16]. It was suggested that carcinomas of the ileal pouch was caused after the chronic pouchitis or the preoperative back wash ileitis [17-19]. In this case, the ileum received a change of the original bacterial flora or a back wash of colonic stool due to the anastomosis. This may have caused the chronic inflammation which lead to the carcinogenesis of the ileum.

## Conclusion

This is a very rare case which showed an intramucosal (Tis) adenocarcinoma of the ileum which originated at the site of the ileosigmoidostomy. This case may provide important information regarding the pathways involved in carcinogenesis of the small intestine.

## Consent

Written informed consent was obtained from the patient for publication of this case report and any accompanying images. A copy of the written consent is available for review by the Editor-in-Chief of this journal.

## Competing interests

The authors declare that they have no competing interests.

## Authors' contributions

SS, ST, SK and TO participated in the surgical resection. HM, MI, MK, and TK carried out the histological examination. All authors read and approved the final manuscript.
